# Characterization of single 1.8-nm Au nanoparticle attachments on AFM tips for single sub-4-nm object pickup

**DOI:** 10.1186/1556-276X-8-482

**Published:** 2013-11-15

**Authors:** Hui-Wen Cheng, Yuan-Chih Chang, Song-Nien Tang, Chi-Tsu Yuan, Jau Tang, Fan-Gang Tseng

**Affiliations:** 1 Department of Engineering and System Science, National Tsing Hua University, 101, Section 2, Kuang-Fu Road, Hsinchu 30013, Taiwan; 2 Institute of Cellular and Organismic Biology, Academia Sinica, Taipei 11529, Taiwan; 3 Medical Image Technology Department, Industrial Technology Research Institute, 195, Section 4, Chung Hsing Road, Hsinchu 31040, Taiwan; 4 Department of Physics, Chung Yuan Christian University, Chungli 32023, Taiwan; 5 Research Center for Applied Sciences, Academia Sinica, 128, Section 2, Academia Road, Taipei 11529, Taiwan

**Keywords:** Au nanoparticle, AFM, Quantum dots, Blinking

## Abstract

This paper presents a novel method for the attachment of a 1.8-nm Au nanoparticle (Au-NP) to the tip of an atomic force microscopy (AFM) probe through the application of a current-limited bias voltage. The resulting probe is capable of picking up individual objects at the sub-4-nm scale. We also discuss the mechanisms involved in the attachment of the Au-NP to the very apex of an AFM probe tip. The Au-NP-modified AFM tips were used to pick up individual 4-nm quantum dots (QDs) using a chemically functionalized method. Single QD blinking was reduced considerably on the Au-NP-modified AFM tip. The resulting AFM tips present an excellent platform for the manipulation of single protein molecules in the study of single protein-protein interactions.

## Background

Scanning tunneling microscopy (STM) [1] and atomic force microscopy (AFM) [2] have revolutionized surface sciences by enabling the study of surface topography and other surface properties at the angstrom-to-micrometer scale. The three major functions of AFM include imaging, spectroscopy (i.e., force-distance curve), and manipulation (nanolithography). AFM techniques employ a very sharp tip as a probe to scan and image surfaces. Spectroscopic information is acquired through forces generated between the tip and the sample when the probe is brought into proximity with the sample surface, according to Hooke's law. Xie et al. [3] classified nanolithographic techniques into two groups: force-assisted and bias-assisted nanolithography.

In AFM, the interactive force between the tip of the probe and the sample surface is determined according to the deflection of a microfabricated cantilever with the tip positioned at the free end. Modifying the probe enables researchers to explore a range of surface characteristics. AFM probes with individual microparticles or nanoparticles attached to the cantilever/tip have been widely used to measure surface forces in AFM and near-field scanning optical microscopy (NSOM) [4] as the geometry and composition of the particle can be well controlled.

Ducker et al. [5,6] were pioneers in the attachment of microspheres to a tipless AFM cantilever with resin. Their colloidal probe technique employed a laser-pulled micropipette attached to an optical microscope. Mak et al. [7] improved this method through their dual wire technique, in which glue and a microsphere are simultaneously applied to a cantilever using two micropipettes. Lantz et al. [8] applied this method to the attachment of FeNdBLa magnetic microparticles to an AFM tip to increase the resolution of magnetic force microscopy. Using a microcolloidal probe, Berdyyeva et al. [9] revealed how the rigidity of human epithelial cells increases with age. Since the 1990s, the microcolloidal probe technique has become one of the most popular techniques for the measurement of surface forces, primarily due to the ease of the technical application, the ability to directly measure forces generated between the particle and various materials, and a more precise contact area than that afforded by a tipless probe. However, the minimum size of particles that can be attached to the AFM tip is approximately 1 μm [10], due mainly to the colloidal attachment process involving optical microscopes and the need to perform micromanipulation with limited resolution. Preventing contamination resulting from the adsorption of glue on the surface of the sphere is crucial to successful attachment.

Ong and Sokolov [11] sought to apply this colloidal attachment method to nanoparticles, by applying glue to the AFM tip; however, this approach resulted in the attachment of many nanoparticles at once. Vakarelski et al. [12,13] developed a wet chemistry procedure to attach a single nanoparticle to the vertex of an SPM probe tip. Wang et al. [14] used an electrochemical oxidation-reduction reaction to attach or grow a nanoparticle (14 ~ 50 nm) selectively on the tip of an AFM probe. Both of these methods employed self-assembled monolayers (SAMs) as material-selective linkers. Okamoto and Yamaguchi [15] employed the photocatalytic effect of a semiconducting material (TiO_2_) to deposit Au nanoparticles (Au-NPs; ranging in size from 100 to 300 nm) to the tip of an AFM cantilever. Unfortunately, controlling the position and size of these nanoparticles proved difficult. Hoshino et al. [16] introduced a nanostamp method to attach sub-10-nm colloidal quantum dot (QD) arrays to a Si probe; however, the number of QDs could not be effectively controlled.

This paper proposes a novel method for picking up individual nano-objects (<4 nm) by directly attaching a 1.8-nm Au-NP to the vertex of an AFM tip without the need for surface modification. The Au-NP is attached through the selective application of short current-limited bias voltage between the Au-NP and the AFM tip. A combination of evaporation and electromigration deposition is used to transfer the Au-NP from the substrate onto the AFM tip in a controllable manner. Direct transmission electron microscopy (TEM) and indirect fluorescence intensity were used to verify that a single 4-nm QD was picked up by the Au-NP-modified AFM probe. This probe is applicable to the manipulation of individual protein molecules.

## Methods

### Materials

The following reagents were used throughout the study: solution of 1.8-nm Au-NP (10 μM of Ni-NTA-Nanogold® in 50 mM MOPs, pH 7.9, Nanoprobes, Yaphank, NY, USA), anhydrous ethanol (≥99.5%, Sigma-Aldrich, St. Louis, MO, USA), 4-nm Qdot® 525 ITK™ amino (PEG) quantum dots (8-μM solution in 50 mM borate, pH 9.0, Invitrogen, Life Technologies, Carlsbad, CA, USA), 16-mercaptohexadecanoid acid (90%, HS(CH_2_)_15_COOH, Aldrich), and deionized (DI) water. *N*-(3-dimethylaminopropyl)-*N*′-ethylcarbodiimide hydrochloride (EDC; Sigma-Aldrich), *N*-hydroxysulfosuccinimide sodium salt (sulfo-NHS; 97%, Aldrich), and phosphate-buffered saline (PBS; pH 7.4, 10×, Invitrogen) were used for bioconjugation.

### Instruments

This study used a NanoWizard® AFM (JPK Instrument, Berlin, Germany), MFP-3D-BIO^TM^ AFM (ASYLUM RESEARCH, Goleta, CA, USA), HITACHI S-4800 field emission scanning electron microscope (FE-SEM; Chiyoda-ku, Japan), JEOL 2000 V UHV-TEM (Akishima-shi, Japan), MicroTime 200 fluorescence lifetime systems with inverse time-resolved fluorescence microscope (PicoQuant, Berlin, Germany), and ULVAC RFS-200S RF Sputter System (Saito, Japan). We also employed 24 mm × 50 mm glass coverslips, a Lambda microliter pipette, and spin coating machine TR15 (Top Tech Machines Co., Ltd., Taichung, Taiwan) for the preparation of samples. Standard silicon polygon-pyramidal tips (Pointprobe® NCH probes, tip radius of curvature <12 nm, resistivity 0.01 ~ 0.025 Ω cm, NanoWorld, Neuchâtel, Switzerland) supported by a cantilever with a spring constant *k* ~ 42 N/m were used for the attachment of Au-NPs. For Au-NP support during the attachment process, we used conductive n-type polished Si (100) wafers (resistivity 0.008 ~ 0.022 Ω cm), purchased from Swiftek Corp. (Hsinchu, Taiwan). An oscilloscope (LeCroy waveRunner 64Xi, 600 MHz, 10 GS/s, Teledyne LeCroy GmbH, Heidelberg, Germany) was used to measure the electric potential. A waveform generator (WW2572A, 250 MS/s, Tabor Electronics, Tel Hanan, Israel) was employed to produce signals on demand.

### Sample preparation (Au-NPs)

A diluted Au-NP solution was prepared by combining the initial Au-NP solution and ethanol at a volume ratio of 1:1,000. Au-NPs were then spread as a monolayer on an n-type silicon wafer by spin-coating. The roughness of the silicon wafer surface had to be sufficiently low (on the order of 100 pm) to ensure that Au-NPs could be imaged using the NanoWizard® AFM.

### Sample preparation (QDs)

A diluted solution of QDs was prepared by combining the initial Qdot® 525 solution with DI water at a volume ratio of 1:10,000. The diluted QD solution was then spread as a monolayer on a glass coverslip by spin-coating. The prepared sample was loaded into a fluorescence microscope.

### Homemade glass/Au film (65 nm)

Half of the 24 mm × 50 mm glass coverslip area was exposed to a sputter source (Au) at a sputter rate of 3 Å/s. AFM images reveal an Au film thickness of 65 nm (see Additional file 1).

### Confocal examination

To provide excitation, a picosecond diode laser (*λ* = 532 nm) was focused on a diffraction-limited spot using an oil-immersion objective lens (N.A. = 1.4, Olympus, Shinjuku-ku, Japan). Fluorescence was collected using the same objective and guided to a confocal pinhole to reject out-of-focus light. After passing through the pinhole, the fluorescence signal was split using a dichroic beam splitter into two beams and then filtered using suitable band-pass filters before being detected by a pair of single-photon avalanche photon diodes. Time-tagged time-resolved (TTTR) measurements were performed during the experiments. TTTR is a time-correlated single-photon counting (TCSPC) technique capable of recording all time-related information for every detected photon, including the relative time between the excitation pulse and photon emission as well as the absolute time between the start of the experiment and the photon emission. We used the TCSPC setup in TTTR mode to monitor the blinking behavior and lifespan of the QDs simultaneously.

## Results and discussion

**Figure 1:**
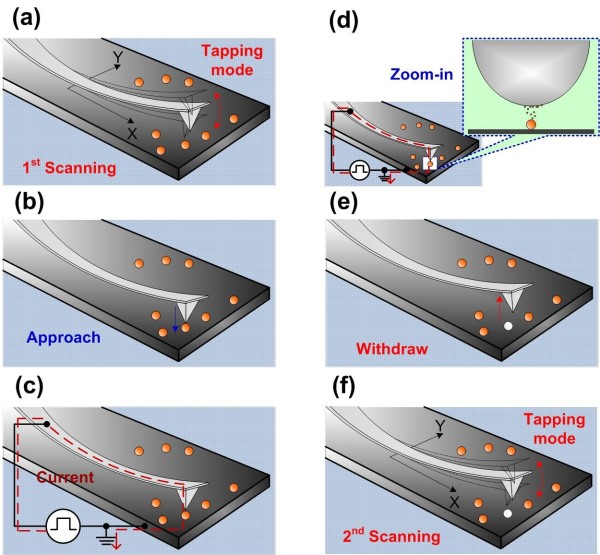
**Schematic diagram depicting the procedures used to attach a single Au-NP to the AFM probe tip. (a)** An image is taken to find the position of each Au-NP. **(b)** The AFM tip is moved above the selected Au-NP. **(c)** The probe is moved toward the Au-NP and the waveform generator applies a pulse of voltage to the AFM probe. **(d)** The Au-NP is evaporated and redeposited on the AFM tip. **(e)** The probe is withdrawn. **(f)** An image is taken again to verify the absence of the Au-NP. The figures are not drawn to scale.

Figure 1 presents a schematic diagram depicting the process of attaching a single Au-NP to the end of an AFM probe. Initially, tapping mode image scanning was performed to determine the position of each Au-NP (Figure 1a). The AFM tip was then moved to a position above the selected Au-NP (Figure 1b). The probe was moved close to the Au-NP; the waveform generator was then used to apply a pulse of voltage to the AFM probe (Figure 1c). In so doing, the Au-NP was evaporated and redeposited on the AFM tip (Figure 1d), whereupon the probe was withdrawn (Figure 1e). Tapping mode image scanning was performed once more to verify the absence of the Au-NP (Figure 1f).

**Figure 2:**
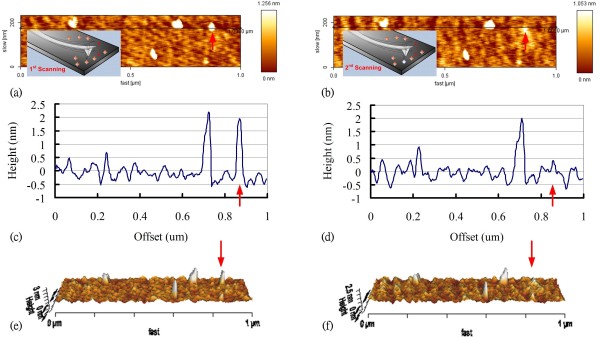
**AFM images, cross sections, and 3D images of the Au-NP.** AFM images of the 1.8-nm Au-NP on Si wafer **(a)** before and **(b)** after the application of a 2-V pulse for 32 ns. **(c)** Cross section following the line in **(a)**. **(d)** Cross section following the line in **(b)**.** (e)** 3D image of **(a)**. **(f)** 3D image of **(b)**. The red arrows indicate the position of the Au-NP before and after the application of 2-V pulse for 32 ns.

AFM images of a 1.8-nm Au-NP before (first scan) and after (second scan) application of the voltage pulse are presented in Figure 2. The second AFM image confirms the transfer of the Au-NP following the application of a 2-V pulse for 32 ns.

In approximately half of the experiments, the AFM images do not reveal obvious differences following the application of the voltage pulse (see Additional file 1). This can be attributed to mechanical drift associated with the AFM [17], resulting in the voltage pulse shifting the position of the selected Au-NP. Another explanation may be that the selected Au-NP was not actually an Au-NP but another nano-object with a height similar to that of the Au-NP.

**Figure 3:**
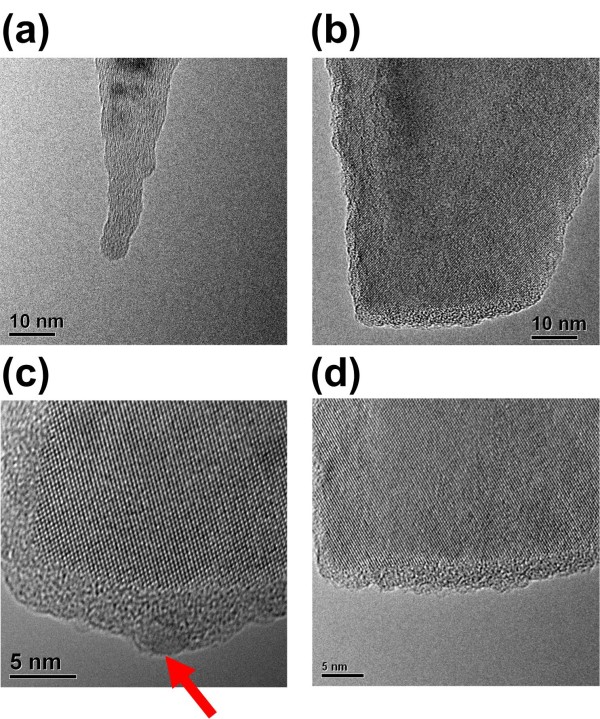
**TEM micrographs of the modified AFM probe. (a)** TEM micrograph of the new AFM probe. **(b)** Following application of a 2-V pulse to the Au-NP for 32 ns, most of the probes presented no visible Au-NP. After conjugating these probes with a QD, **(c)** 44% of tips were able to pick up single QDs (red arrow) and **(d)** 56% of tips were unable to pick up anything.

To further verify the attachment of the Au-NP to the probe, we examined TEM micrographs of the modified AFM probe, as shown in Figure 3. To facilitate comparison, a new probe was also imaged. The original tip radius of curvature was verified as less than 8 nm (Figure 3a). In a series of experiments (using more than 50 AFM probes) and the same voltage pulse of 2 V for 32 ns, we were unable to observe Au-NPs on most of the AFM tips (Figure 3b), suggesting either that the Au atoms were distributed on the AFM tip without any particular structure or that they did not attach. In a few cases, we observed complete Au-NPs on the AFM tips in TEM micrographs; however, these Au-NPs appear to have been adsorbed on the AFM tips randomly [18] (see Additional file 1 for details). We then conducted conjugation experiments using 4-nm QDs to verify the existence of Au on these tips. TEM micrographs demonstrated that 44% of the tips succeeded in picking up single QDs at the vertex (Figure 3c), while the remaining 56% did not (Figure 3d).

**Figure 4:**
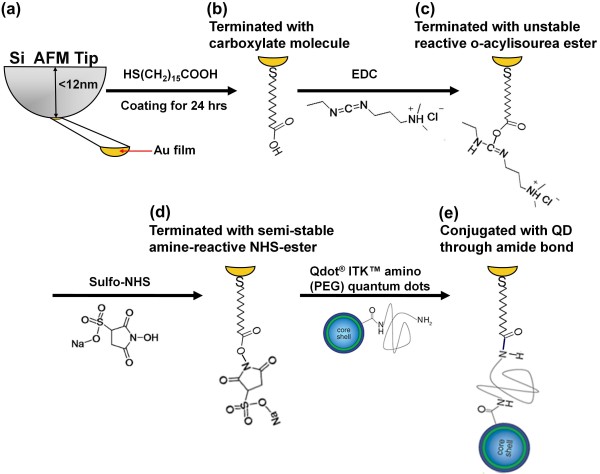
**Process of conjugation between Au-NP and a 4-nm QD. (a, b)** HS(CH_2_)_15_COOH is first self-assembled on the Au atoms at the AFM tip to expose the carboxylic acid functional group. **(c, d)** Reaction with EDC and sulfo-NHS to form amine-reactive ester. **(e)** Attachment of functionalized QDs by an amide bond.

Figure 4 illustrates the process of conjugating the Au-NP with QDs. HS(CH_2_)_15_COOH was first self-assembled on the Au atoms at the AFM tips to expose the carboxylic acid functional group (Figure 4a,b) for further QDs conjugation. Following activation by EDC and sulfo-NHS, an amine-reactive ester formed (Figure 4c,d). Finally, Qdot® ITK™ amino (PEG) QDs conjugated with the Au-NP through the formation of an amide bond.

**Figure 5:**
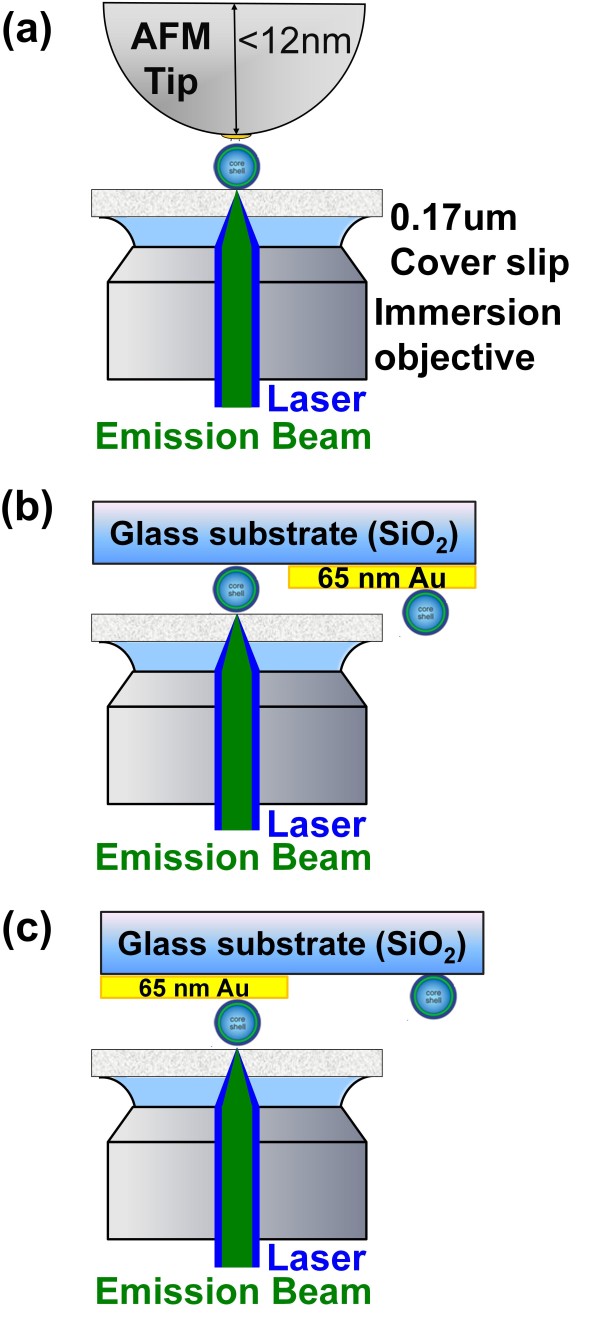
**Experimental setup for observation of fluorescence intensity in single QDs. (a)** Conjugated with the Au-NP-modified AFM probe, **(b)** on the glass portion of the reference sample, and **(c)** on the Au film portion of the reference sample. All measurements were performed in a dark compartment at room temperature.

**Figure 6:**
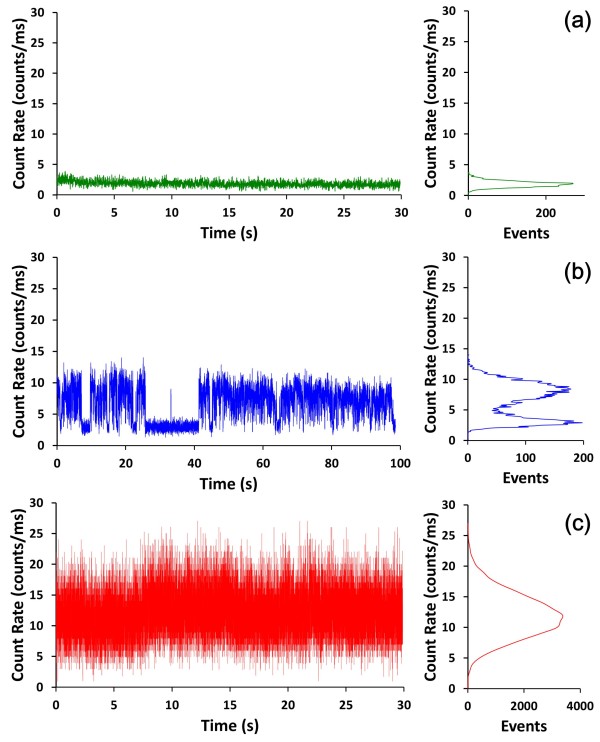
**Typical fluorescence intensity trajectories of single QDs.** On the **(a)** Au-NP-modified AFM probe, **(b)** glass surface, and **(c)** 65-nm Au film.

To verify the existence of a single QDs on the AFM tip, we monitored the fluorescence of single QDs using a far-field laser scanning confocal microscope. For comparison, we prepared half-glass and half-Au film (65 nm) substrates as reference samples (Figure 5). QDs samples were prepared by spin-coating a 0.1-nM solution of QD525 on the glass/Au film (65 nm) substrates. The root-mean-squared (RMS) value of the surface roughness on the Au film was estimated at less than 10 nm (see Additional file 1). The resulting emission trajectories are presented in Figure 6.

The photoblinking phenomenon, or fluorescence intermittency, is an important characteristic of QDs [19]. The term refers to the temporal disappearance of emitted light when molecules or particles undergo reversible transitions between ‘on’ and ‘off’ states. Single QDs on glass clearly demonstrate this phenomenon, leading to bimodal variations in intensity (Figure 6b).

This study demonstrated that through the appropriate coupling of Au-NP to the modified AFM probe, single QDs exhibit suppressed blinking and quenched fluorescence intensity (approximately 2-fold) (Figure 6a). Single QDs on the 65-nm Au film (Figure 6c) also exhibited suppressed blinking behavior; however, fluorescence intensity was increased (approximately 1.5-fold). Applying QDs on a 10-nm Au film surface resulted in the enhancement of fluorescence intensity approximately 3-fold (see Additional file 1). These results support those of previous studies, in which the intensity of photoluminescence is either enhanced or quenched on roughened and smooth metal surfaces [20-25], respectively. However, conjugating QDs to the Au-NP modified-AFM probe presented a slightly different situation, which may be attributed to the effect of the nanoenvironment associated with the QD. These results are similar to those of Ratchford et al. [26] and Bharadwaj and Novotny [27]. In these studies, an Au-NP was pushed proximal to a CdSe/ZnS QDs resulting in the quenching of fluorescence intensity (approximately 2.5-fold [26] and approximately 20-fold [27], respectively). Our results provide evidence of the existence of a small Au-film on the AFM tip.

### Mechanism: evaporation and electromigration

One possible mechanism involved in the attachment of a 1.8-nm Au-NP to an AFM tip under a pulse of electrical voltage may be the evaporation and electromigration of Au-NPs induced by the strong electric field, resulting in a small area of Au film coating the AFM tip (an Au film roughly 4 nm in diameter coating the tip without a visible Au particle).

In this scenario, an Au-NP is melted and attracted to the tip apex through a sudden increase in the electric field due to a voltage pulse. Au has a vapor pressure of 10^-5^ Torr (estimated from bulk Au and is presumably lower for Au nanoparticles). As a result, Au is first evaporated and the Au vapor is then guided by the electrical field between the AFM apex and the substrate to be deposited over a limited region of the AFM apex. The energy required to transfer Au vapor is very small and can be disregarded.

Throughout the Au-NP evaporation process, the energy supplied to the system can be estimated as *i*_0_*V*_s_*t*. According to the experimental setup and measurements (see Additional file 1), the values of *i*_0_, *V*_s_, and *t* were 3 × 10^-6^ A, 2-V, and 32 ns, respectively. Equation 1 derives input energy *E*_i_:

(1) $$E_{i}=i_{0}V_{s}t=4\times {10^{-}}^{14}J.$$

The minimum required energy *E*_m_ is that required to melt the Au-NP and heat the Si tip to the melting temperature of Au. The thermal energy required to melt the Au-NP is *m*_Au-NP_*C*_P,Au_ (*T*_m,Au-NP_ – *T*_0_), where *m*_Au-NP_ is the mass of the 1.8-nm Au-NP, *C*_P,Au_ ≈ 129 J/(kgK) is the specific heat capacity of Au, *T*_m,Au-NP_ is the melting temperature of the 1.8-nm Au-NP, and *T*_0_ ≈ 298 K is the room temperature [28].

To calculate the mass of Au, we estimated the number of Au atoms in a nanoparticle. Cortie and Lingen [29] pointed out that the atomic packing density of nanogold is approximately 0.70 (between bcc and fcc). There are about 171 Au atoms in a 1.8-nm Au-NP and *m*_Au-NP_ = 2.14 × 10^-27^ kg (*ρ*_Au-NP_ ≈ *ρ*_Au_ = 19,300 kg/m^3^).

Experimental, theoretical, and computer-simulated studies have shown that melting temperature depends on cluster size [29]. These studies suggest a relationship of temperature dependence defined by the following: *T*_m_ = *T*_b_ – *c* / *R *[30], where *T*_m_ is the melting temperature of a spherical nanoparticle of radius *R*, *T*_b_ is the bulk melting temperature, and *c* is a constant. From the literature, *T*_m,Au-NP_ ≈ 653 K. Thus, *m*_Au-NP_*C*_P,Au_ (*T*_m,Au-NP_ – *T*_0_) = 9.8 × 10^-23^ J.

The thermal energy required to heat the apex of the tip to *T*_m,Au-NP_ is *m*_apex_*C*_P,Si_ (*T*_m,Au-NP_ – *T*_0_), where *m*_apex_ is the estimated mass of the spherical Si tip apex and *C*_P,Si_ ≈ 712 J/kg/K is the specific heat capacity of Si [28]. The mass of the Si probe to be heated is estimated according to its spherical volume with a radius equivalent to the curvature of the tip (12 nm). As a result, *V*_apex_ = 7.24 × 10^-24^ m^3^, *ρ*_Si_ = 2,330 kg/m^3^, and *m*_apex_*C*_P,Si_ (*T*_m,Au-NP_ – *T*_0_) = 4.27 × 10^-15^ J.

Assuming an adiabatic system (this process occurs in less than 40 ns; therefore, this assumption is reasonably accurate), the minimum required energy *E*_m_ can be estimated using Equation 2:

(2) $$\begin{array}{l} E_{m}=m_{\mathrm{Au}-\mathrm{NP}}C_{P,\mathrm{Au}}\left(T_{m,\mathrm{Au}-\mathrm{NP}}–T_{0}\right)+m_{\mathrm{apex}}C_{P,\mathrm{Si}}\left(T_{m,\mathrm{Au}-\mathrm{NP}}–T_{0}\right)\\ \;=4.27\times {10^{-}}^{15}J. \end{array}$$

The minimum required energy (*E*_m_, Equation 2) is roughly 1 order of magnitude lower than that of the supplied energy (*E*_i_, Equation 1), suggesting that sufficient input energy exists to melt the Au-NPs. This is a reasonable range and can be adjusted through manipulation of the current *i*_0_, *m*_apex_, and *m*_Au-NP_.

**Figure 7:**
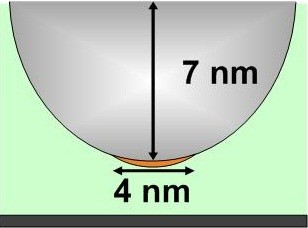
Schematic diagram showing distribution of Au atoms on the tip apex.

We propose a model of a single-atom layer of Au film formed on the apex of the AFM tip in order to estimate the maximum deposition area by the evaporated Au, as shown in Figure 7. An actual AFM tip image is presented in Figure 3b with no Au-NPs visible on the AFM tip. We estimated that there are roughly 171 Au atoms in a 1.8-nm Au-NP. If these Au atoms were packed closely together, the total area occupied could be estimated as 1,145 Å^2^ (from the 1.46 Å of a single Au atom radius), resulting in a circle with diameter of approximately 4 nm. This area would be small enough for our prospective single-QDs modification experiment.

In Figure 3b, a very small portion of the AFM tip presents a lattice darker than the rest of the Si tip. The tip curvature in this area is greater than that in the new tip. We can deduce from this that Si atoms at the tip surface underwent reflow under the electric field. At the same time, the Au-NP melted, evaporated, and formed a compound with the Si at the tip apex. The dark lattice area is estimated to be 1,000 Å^2^, which is very close to the circular ‘Au-atom-layer’ deposition area (1,145 Å^2^) predicted by the evaporation, electromigration, and deposition model. This case represents 44% of all the Au-NP attachment cases.

## Conclusions

This study presents a novel AFM probe modification scheme in which a 1.8-nm Au-NP is applied by means of a current-limited voltage pulse (2 ~ 5 V, ≥32 ns). TEM micrographs and fluorescence inspection results prove the existence of an Au-NP on the apex of the probe. An experiment involving the conjugation of single QDs also demonstrated the existence of a small amount of Au (equal to or less than 4 nm in diameter) deposited on the AFM tips, as well as the ability of the Au-modified AFM tip to pick up single macromolecules (QDs). We also discuss the mechanisms that may be involved in Au attachment: evaporation, electromigration, and deposition. The Au-NP was melted, evaporated, and deposited onto the tip apex by a sudden increase in the electric field due to a voltage pulse. The resulting AFM tips present an excellent platform for the manipulation of single protein molecules in the study of single protein-protein interactions.

## Competing interests

The authors declare that they have no competing interests.

## Authors' contributions

FGT conceived of the research work and participated in the analysis. YCC performed the TEM analysis. SNT participated in the bias-applying circuit, coordination, and analysis. CTY and JT performed the fluorescence intensity inspection design and analyses. HWC performed all AFM experiments, analyzed the TEM and fluorescence results, and drafted the manuscript. All authors have read and approved the final manuscript.

## Supplementary Material

Additional file 1 **The file contains the method for the measurement of *I*, *V*, and *R*; failed experiments; adhesion of an Au-NP to the probe apex during scanning; and experimental setup for fluorescence inspection.** Click here for file
