# Supplementary material for: Characterization of single 1.8-nm Au nanoparticle attachments on AFM tips for single sub-4-nm object pickup
Source: Nanoscale Res Lett. 2013 Nov 15;8(1):482. doi: 10.1186/1556-276X-8-482 (PMC3835868; doi:10.1186/1556-276X-8-482)
Supplement: Additional file 1 — The file contains the method for the measurement of I, V , and R; failed experiments; adhesion of an Au-NP to the probe apex during scanning; and experimental setup for fluorescence inspection. [file 1556-276X-8-482-S1.docx]

**Supporting information** for Manuscript entitled “*Characterization of single 1.8 nm Au-nanoparticle attachments on AFM tips for single sub-4 nm object pick-up*” by H. W. Cheng, Y. C. Chang, S. N. Tang, C. T. Yuan, J. Tang, and F. G. Tseng^＊^

**Method for the measurement of *I*, *V* and *R***


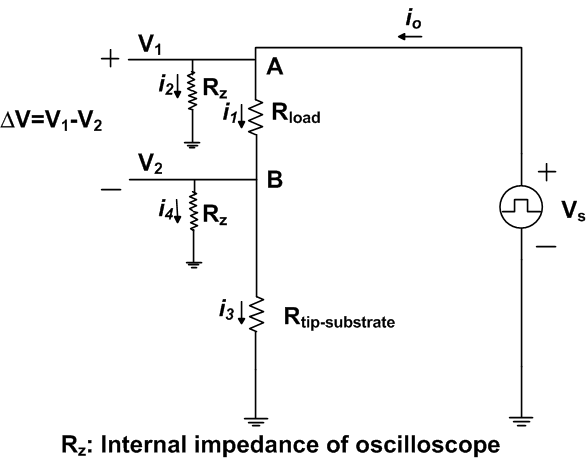


Figure 1. Experimental setup for measuring point contact resistance between tip and substrate.

To determine the appropriate bias voltage range and current limitations, we set up the experiment shown in Figure 1 to measure point contact resistance between the tip and substrate (*R*_tip-substrate_). All experiments were conducted under atmosphere with 50% relative humidity at 25°C. First, the AFM tip was moved toward the Au-NP using the NanoWizard^®^ AFM. A voltage pulse *V*_s_ (with a duty cycle *t*) from a waveform generator was applied between *R*_load_ (known) and *R*_tip-substrate_ (unknown), providing total current *i*_0_ to the circuit. An oscilloscope (with high internal impedance *R*_z_) was then employed to measure the voltage at positions A (*V*_1_) and B (*V*_2_). Kirchhoff's Law [Equations (1) ~ (4)] was used to calculate *R*_tip-substrate_. After many trials, we determined the appropriate *R*_load_ (3 MΩ) and *R*_tip-substrate_ (~ 5 MΩ).

{[(*R*_tip-substrate_ // *R*_z_) ＋ *R*_load_] // *R*_z_ } × *i*_0_ = *V*_s_ (1)

In the above equation, *i*_0_, *V*_s_ and *i*_0_*V*_s_*t* are the total current, the applied voltage and the total energy supplied by the waveform generator, respectively.

Equation (1) can be rewritten as follows:

$\left[\left(\frac{1}{R_{\text{tip-substrate}}}+\frac{1}{R_{\text{z}}}\right)+R_{\text{load}}\right]^{-1}+\frac{1}{R_{\text{z}}}=\frac{V_{\text{s}}}{i_{o}}$ (2)

The relationship between the currents is as follows:

*i*_0_ = *i*_1_ ＋ *i*_2_ = (*i*_3_ ＋*i*_4_) ＋*i*_2_ (3)

$i_{o}=\frac{\Delta V}{R_{\text{load}}}+\frac{\Delta V}{R_{\text{z}}}=V_{2}\left(\frac{1}{R_{\text{tip-substrate}}}+\frac{1}{R_{\text{z}}}\right)+\frac{\Delta V}{R_{\text{z}}}$ (4)

*R*_z_, *R*_load_ and Δ*V* are known values; therefore, the unknown values *R*_tip-substrate_ and *i*_0_ can be calculated from Equations (2) and (3).

To attach an Au-NP onto the AFM cantilever tip vertex, we applied voltage pulses from 16 mV to 16 V with pulse widths ranging from 16 ns to 1 us. Following the application of each voltage pulse, the apex of the AFM tip was inspected using FE-SEM. It is worth noting that the high-energy-focused electron-beam of the SEM can damage the profile of the tip apex. However, this damage can be alleviated by decreasing the acceleration voltage from 15 kV to 5 kV. Figure 2 presents SEM images of the tip profiles following the application of various voltage pulses. As shown in Figure 2a, the profile of the tip apex remained sharp when the pulse voltage was below 5 V. When the pulse voltage exceeded 6 V, the tip apex was blunted, as shown in Figure 2b. Therefore, the amplitude of the output voltage from the waveform generator was controlled to below 5 V for the remainder of the experiments.


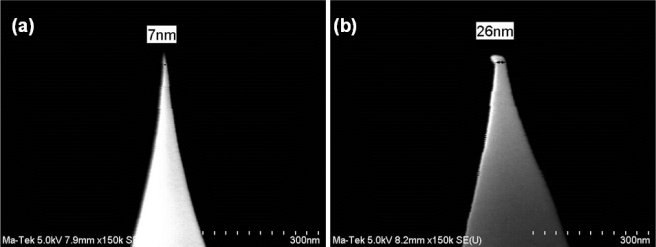


Figure 2. SEM images of tip vertex profile following application of (a) 5 V and (b) 16 V DC

**Failed experiments**

In approximately half of our experiments, the AFM images fail to reveal an obvious difference following the application of the voltage pulse (Figure 3). This can be attributed to the mechanical drift of the AFM, resulting in the voltage pulse shifting the position of the selected Au-NP. Prior to the application of the pulse, the Au-NP was located at x ~ 2.2 um and y ~ 0.5 um (Figure 3a). Following the application of the pulse, the Au-NP was located at x ~ 2.3 um and y ~ 0.6 um (Figure 3b). Another reason for the failed attempts may be that the selected Au-NP was not actually an Au-NP but another nano-object with the same height as Au-NP.

Figure 3. AFM images of a 1.8 nm Au-NP on a Si wafer: (a) before and (b) after applying a 32 ns 2 V pulse to the particle indicated by the arrow; (c) cross-section following the line in (a); (d) cross-section following the line in (b); (e) 3D image of (a); (f) 3D image of (b)

**Adhesion of an Au-NP to the probe apex during scanning**

Au-NPs can also be attached to the AFM tip by the adhesion force between the Au-NP and the AFM tip during scanning, as shown in Figure 4. Attachment of the Au-NP to the AFM tip may occur when the adhesion force between the tip and the Au-NP is greater than that between the Au-NP and the substrate. The main components of the adhesion force are capillary, van der Waals and electrostatic forces. JiaPeng et al. [1] observed that a small number of Au-NPs could be picked up even at zero bias, which is likely due to van der Waals or capillary-force interactions between the AFM tip and the Au-NPs on the substrate surface.

Figure 4. (a) TEM micrograph of probe with a single 1.8 nm Au-NP on the vertex; (b) magnification TEM image of (a); (c) probe with two 1.8 nm Au-NPs on the vertex.

**Experimental setup for fluorescence inspection**

Figure 5. Experimental setup for observation of the QDs conjugated on the Au-NP modified AFM tip. The center of AFM tip is aligned with the center of the laser focus of a confocal microscope.

Figure 6. Experimental setup for observation of the QDs on the coverslip and reference sample (half-glass and half-Au surface).

Figure 7. Half-glass and half-Au reference sample prepared for this study: (a) AFM image at boundary of sample; (b) cross-section following the line in (a); (c) 3D image of (a); (d) schematic diagram of reference sample.

Figure 8. Typical fluorescence intensity trajectories of single QDs on 10 nm Au film. The blinking of QDs was suppressed on rough Au and the intensity increased ~3 fold.

References

1. Xu J, Kwak KJ, Lee JL, Agarwal G: **Lifting and sorting of charged Au nanoparticles by electrostatic forces in atomic force microscopy.** *Small* 2010, **6:**2105–2108.
